# SPIDEN: deep Spiking Neural Networks for efficient image denoising

**DOI:** 10.3389/fnins.2023.1224457

**Published:** 2023-08-11

**Authors:** Andrea Castagnetti, Alain Pegatoquet, Benoît Miramond

**Affiliations:** Université Côte d'Azur, CNRS, LEAT, Sophia Antipolis, France

**Keywords:** denoising, Spiking Neural Networks, quantization error, low latency, sparsity, direct training, energy consumption

## Abstract

In recent years, Deep Convolutional Neural Networks (DCNNs) have outreached the performance of classical algorithms for image restoration tasks. However, most of these methods are not suited for computational efficiency. In this work, we investigate Spiking Neural Networks (SNNs) for the specific and uncovered case of image denoising, with the goal of reaching the performance of conventional DCNN while reducing the computational cost. This task is challenging for two reasons. First, as denoising is a regression task, the network has to predict a continuous value (i.e., the noise amplitude) for each pixel of the image, with high precision. Moreover, state of the art results have been obtained with deep networks that are notably difficult to train in the spiking domain. To overcome these issues, we propose a formal analysis of the information conversion processing carried out by the Integrate and Fire (IF) spiking neurons and we formalize the trade-off between conversion error and activation sparsity in SNNs. We then propose, for the first time, an image denoising solution based on SNNs. The SNN networks are trained directly in the spike domain using surrogate gradient learning and backpropagation through time. Experimental results show that the proposed SNN provides a level of performance close to the state of the art with CNN based solutions. Specifically, our SNN achieves 30.18 dB of signal-to-noise ratio on the Set12 dataset, which is only 0.25 dB below the performance of the equivalent DCNN. Moreover we show that this performance can be achieved with low latency, i.e., using few timesteps, and with a significant level of sparsity. Finally, we analyze the energy consumption for different network latencies and network sizes. We show that the energy consumption of SNNs increases with longer latencies, making them more energy efficient compared to CNNs only for very small inference latencies. However, we also show that by increasing the network size, SNNs can provide competitive denoising performance while reducing the energy consumption by 20%.

## 1. Introduction

Image denoising algorithms are intensively used in smartphones and embedded vision systems to recover image quality by reducing the amount of noise of the raw image. Image denoising performance have increased during the last few years and recent methods based on Deep Convolutional Neural Networks (DCNNs) have provided very high scores (Zhang et al., 2017) to the point of outreaching classical spatial and patch-based algorithms (Gu et al., 2014). However, deploying AI-based algorithms on embedded devices poses many problems. The limited amount of memory available, the power consumption and thermal dissipation are indeed critical for embedded battery powered platforms. Therefore, the deployment of AI-based solutions on mobile devices requires a careful adaptation of the neural network architecture to the restrictions of AI hardware in mobiles. Such optimizations can include network pruning (Guo et al., 2021), low-bit quantization (Esser et al., 2020; Yamamoto, 2021; Young et al., 2021) or platform-aware neural architecture search (Wu et al., 2019; Kim et al., 2022). Specifically low-bit quantization allows reducing the precision needed to represent neuron's activations and network parameters, i.e., weights and biases, thus reducing the computation and memory requirements while minimizing the accuracy loss compared to a full-precision Artificial Neural Networks (ANN).

Meanwhile, Spiking Neural Networks (SNNs) are emerging as an alternative for the design of low-power processing hardware (Abderrahmane et al., 2020). The spike information coding used by SNNs enables sparse and event-based computation through the network. Moreover, relying on a temporal binary code allows to replace the multiply-accumulate (MAC) operations with simpler and more energy-efficient accumulation (ACC) operations. Both quantized ANNs and SNNs make use of low precision, i.e., quantized, representations of the neurons activations to reduce the computational requirements. Specifically, spiking neurons act like quantizers by discretizing the input signal into a stream of spikes (Li et al., 2022; Castagnetti et al., 2023b). The quantization error of the signal reconstructed from the spike train is directly related to the latency of the network. Thus, increasing the conversion time lowers the quantization errors but at the cost of energy overhead. A trade-off also exists for quantized ANN, where the bitwidth used to represent the neuron activations has an effect on the accuracy, the computational and then the energy costs.

In this paper we address the problem of training efficient SNN's for image denoising. This regression task is challenging since the network has to predict a continuous value (i.e., the noise amplitude) for each pixel of the image with high precision. We choose to train the SNN directly in the spiking domain using surrogate gradient learning (Neftci et al., 2019). This allows us to adapt the neuron parameters during training. We are then able to simultaneously optimize the task loss and the quantization noise introduced by the spiking neurons. Using our training strategy, we achieve performance results very close to a full-precision ANN while minimizing the overall latency of the SNN. Finally, we compare the resulting SNNs with quantized ANN both in terms of performance and energy consumption.

Here, are the key contributions of this paper:

- **Direct SNN training with learnable quantization**: The discretization introduced by spiking neurons limits the performance of SNN by introducing quantization noise. By taking into account the quantization noise during training we show that we can improve the performance while reducing the latency of the SNN.
- **Efficient SNN for image denoising**: Our approach is validated on Gaussian image denoising and compared against full-precision and quantized ANN. Specifically, our SNN achieves a signal-to-noise ratio which is only 0.25 dB below the performance of the equivalent full-precision ANN.
- **Energy efficiency estimation and comparison between SNNs and quantized-ANNs**: Our SNN is compared against an ANN using a metric for energy consumption estimation that takes into account synaptic operations, memory accesses and element addressing. We analyze the energy consumption for different network latencies and network sizes. We show that the energy consumption of SNNs increases with longer latencies, making them more energy efficient compared to CNNs only for very small inference latencies. However, we also show that by increasing the network size, SNNs can provide competitive denoising performance while reducing the energy consumption by 20%. These results will be summarized in Section 4.7.

## 2. State of the art

Spiking Neural Networks (SNNs) are biologically-inspired models of neural networks. SNNs are stateful systems, their internal state is represented by the value of the membrane potential of the spiking neurons that compose the network. Moreover, SNNs use spikes to encode and communicate information. Since a single spike can only represent a binary value, spiking neurons have to generate sequences of spikes to encode and communicate complex information. The time required to encode the information represents the latency of the SNN. Reducing the inference latency of SNNs (i.e., the number of timesteps), without degrading the performance, is an active area of research since it is crucial to obtain the energy saving promised by the SNNs.

Early works demonstrated the feasibility of converting full precision ANNs to SNNs by matching the firing rate of the neurons (Diehl et al., 2015; Sengupta et al., 2019). With these approaches it is almost possible to obtain a lossless conversion at the cost of a long integration time (hundreds to thousands of timesteps) on complex image classification tasks, e.g., CIFAR-10/100 or Imagenet.

Recent works (Li et al., 2021; Guo et al., 2022b) have shifted the attention from firing rate matching to the analysis of the quantization process carried out by the spiking neurons. Li et al. (2021) propose a post-training calibration pipeline to fine-tune the parameters of an ANN, in order to reduce the quantization error, before transferring the weights to the SNN. They were able to reduce the inference latency to only few timesteps but at the cost of a significant reduction in performance. In Bu et al. (2022), the authors propose an ANN to SNN conversion technique that initializes the membrane potential of the spiking neurons in order to minimize the quantization error. A significant performance drop is observed on complex image classification tasks (e.g., CIFAR10/100) when the network latency is lower than 16 timesteps. Besides image classification, ANN to SNN conversion has also been applied to challenging tasks like object detection (Kim et al., 2020) which require high numerical precision in predicting the output values of neural networks. Here, the authors propose a channel-wise weight normalization technique to reduce the quantization error of the spiking neurons. The proposed network, Spiking-YOLO, can match the performance of the equivalent ANN implementation but at the cost of a high inference latency since thousands of timesteps must be used. Optimizing network performance and quantization noise independently, like in the post-training calibration methods described above, results in sub-optimal solutions, thus explaining the drop of performance or the extremely high latency required to match the ANN performance.

Li et al. (2022) propose to use Quantization-Aware-Training (QAT) to train a quantized ANN before transferring the weights to the SNN. The quantization mapping is learned using the Learned Step Size Quantization (LSQ) algorithm (Esser et al., 2020) during training while minimizing the task loss. The resulting SNN takes advantage of the joint optimization carried out by QAT, thus providing high accuracy and low latency.

Besides ANN-to-SNN conversion, new training methods have recently emerged as an alternative to train SNN directly in the spike domain (Neftci et al., 2019). To do so, the non-differentiable part of the spiking neuron is replaced during back-propagation by a surrogate function. This makes possible to compute an approximation of the gradient for SNNs, that can be trained using back-propagation-through-time (BPTT) as for standard recurrent networks. Deng et al. (2022) propose Temporal Efficient Training (TET), which is based on direct SNN training with surrogate gradient (SG). TET constrains the network output at each timesteps to be close to the target distribution thus improving the training process and reducing the generalization error. Guo et al. (2022a) introduce the Information maximization loss (IM-Loss) to increase the information flow between the membrane potential and the spiking output of the neurons, thus reducing the quantization error of the spiking neurons. In Guo et al. (2022c), the authors propose a composite loss to constrain the membrane potential distribution of the spiking neuron. The loss term indeed penalizes the shift of membrane potential distribution outside of the range [0, *V*_*th*_]. By matching the membrane potential distribution with the conversion range of the spiking neuron, the quantization error is reduced and the accuracy improves. Moreover, the proposed method helps alleviate the exploding/vanishing gradients problem, thus improving the convergence as well. The authors of Wang et al. (2022) propose a learnable thresholding mechanism with a moderate dropout method (LTMD) to enhance the learning of SNNs. The SNN network is trained using surrogate gradient and back-propagation. During training both synaptic connections, i.e., weights and biases, as well as neurons threshold can be updated. The proposed SNNs can achieve better accuracies for both static and neuromorphic test datasets with fewer timesteps. Similarly, Castagnetti et al. (2023b) have shown that it is possible to achieve low latency SNNs for complex image classification tasks with a limited accuracy drop compared to full-precision ANN. The authors propose to match the conversion range of the spiking neuron to the membrane potential distribution by learning the *V*_*th*_ parameter.

Most of the previous works targeted image classification problems. Regression problems and specifically spiking autoencoders have only been sparsely explored. Comşa et al. (2021) introduced a spiking autoencoder to reconstruct images with high fidelity using temporal coding. The SNN is trained using surrogate gradients and standard back-propagation. The architecture of the spiking autoencoder is limited to a single hidden-layer network and is tested on low resolution images (MNIST, FMNIST). Similarly Roy et al. (2019) proposed a spiking autoencoder for image reconstruction trained using surrogate gradient and BPTT. The autoencoder is composed of a 3-layer fully-connected network and is also tested on the same low-resolution datasets. The previous works only consider shallow networks and low-resolution datasets thus making difficult to compare these results with state of the art non-spiking autoencoders for image denoising (Zhang et al., 2017).

To the best of our knowledge this work is first study on spiking deep convolutional autoencoder for image denoising. Our work is inspired by the convolutional architecture proposed in Zhang et al. (2017) and is tested on moderately high-resolution (256 × 256 and 512 × 512 pixels) images that are widely used for the evaluation of Gaussian denoising methods. In the following section, we analyze the information coding of spiking neurons and we characterize the quantization noise. Based on this analysis we then propose a trainable quantization scheme for spiking neurons in Section 3.2.

## 3. Methods

### 3.1. Information coding with spiking neurons

In this section, we characterize the quantization function of a spiking neuron with respect to its parameters. The goal is to show how the properties of the input (e.g., the range), the conversion time and the parameters of the neuron affect the conversion process. We restrict our analysis to the Integrate-and-Fire with soft-reset spiking neuron. This neuron model, which is widely used in SNN research (Li et al., 2022; Castagnetti et al., 2023b) implements a uniform quantization scheme which leads to a reduced quantization error compared to other spiking neuron models.

**Figure 1:**
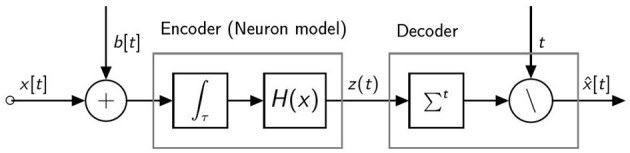
Information coding pipeline for a spiking neuron with a single pre-synaptic input.

The information coding pipeline for a spiking neuron with a single pre-synaptic input is shown in Figure 1. The neuron receives a pre-synaptic input current which is composed of two terms: *x*[*t*] which depends on the activity of the pre-synaptic neurons and a bias *b*[*t*]. The neuron converts the input current into a train of spikes represented by *z*[*t*]. The spiking output is decoded using rate coding. The output of the decoder, $\hat{x}\left[t\right]$ is therefore a quantized version of the input. By considering a constant input current, that is *x*[*t*] = *x*, ∀*t* and *b*[*t*] = *b*, ∀*t*, it can be shown (Castagnetti et al., 2023b) that, for this specific neuron model, the neuron output and the rate decoded output can be approximated as follows:


(1) $$\begin{array}{ll} \overset{T}{\underset{t=1}{\sum }}z\left(t\right) & =\min\left\{T,\left\lfloor \frac{\left(x+b\right)T}{V_{th}}\right\rfloor \right\}={\left\lfloor \frac{\left(x+b\right)T}{V_{th}}\right\rfloor }_{T} \end{array}$$

(2) $$\begin{array}{ll} \hat{x}\left(x,T,V_{th},b\right) & =\frac{1}{T}\overset{T}{\underset{t=1}{\sum }}z\left(t\right)=\frac{1}{T}{\left\lfloor \frac{\left(x+b\right)T}{V_{th}}\right\rfloor }_{T} \end{array}$$

From Equation (2), we can observe that the output $\hat{x}$ depends both on the neuron parameters (*V*_*th*_ and the integration time *T*) and on the inputs *x* and *b*. In the following we study the effect of these parameters, especially on the quantization error introduced by the spiking neuron, that is the Signal-to-Quantization-Noise-Ratio (SQNR) defined below:


(3) $$\begin{array}{l} SQNR\left(x\right)=10\log_{10}\left(\frac{𝔼\left[x^{2}\right]}{𝔼\left[{\left(x-\hat{x}\right)}^{2}\right]}\right) \end{array}$$

We assess the quantization performance of the spiking neuron by encoding and decoding a set of images (Set12), from the Berkeley segmentation dataset (Roth and Black, 2005).

In our setup the spiking neurons are used as scalar quantizers, that is each pixel of the images is encoded using a different neuron. Their outputs are then decoded to reconstruct the pixels and compute the average SQNR for each image of the test set. We set *V*_*th*_ = 1 for each neuron. We repeat the quantization process for a different amount of timesteps, specifically *T*∈[1, 32]. To show the effect of the input range on the conversion process, the pixels of the images are rescaled to the range [0, *x*_*max*_], where *x*_*max*_∈[0.25, 2]. For each set of parameters [*V*_*th*_, *T, x*_*max*_], we measure the SQNR and the average amount of spikes, that we call θ, generated by each neuron during the conversion process:


(4) $$\begin{array}{l} \theta =\frac{\overset{n}{\underset{i=0}{\sum }}\overset{m}{\underset{j=0}{\sum }}z_{i,j}\left(t\right)}{n\times m} \end{array}$$

**Figure 2:**
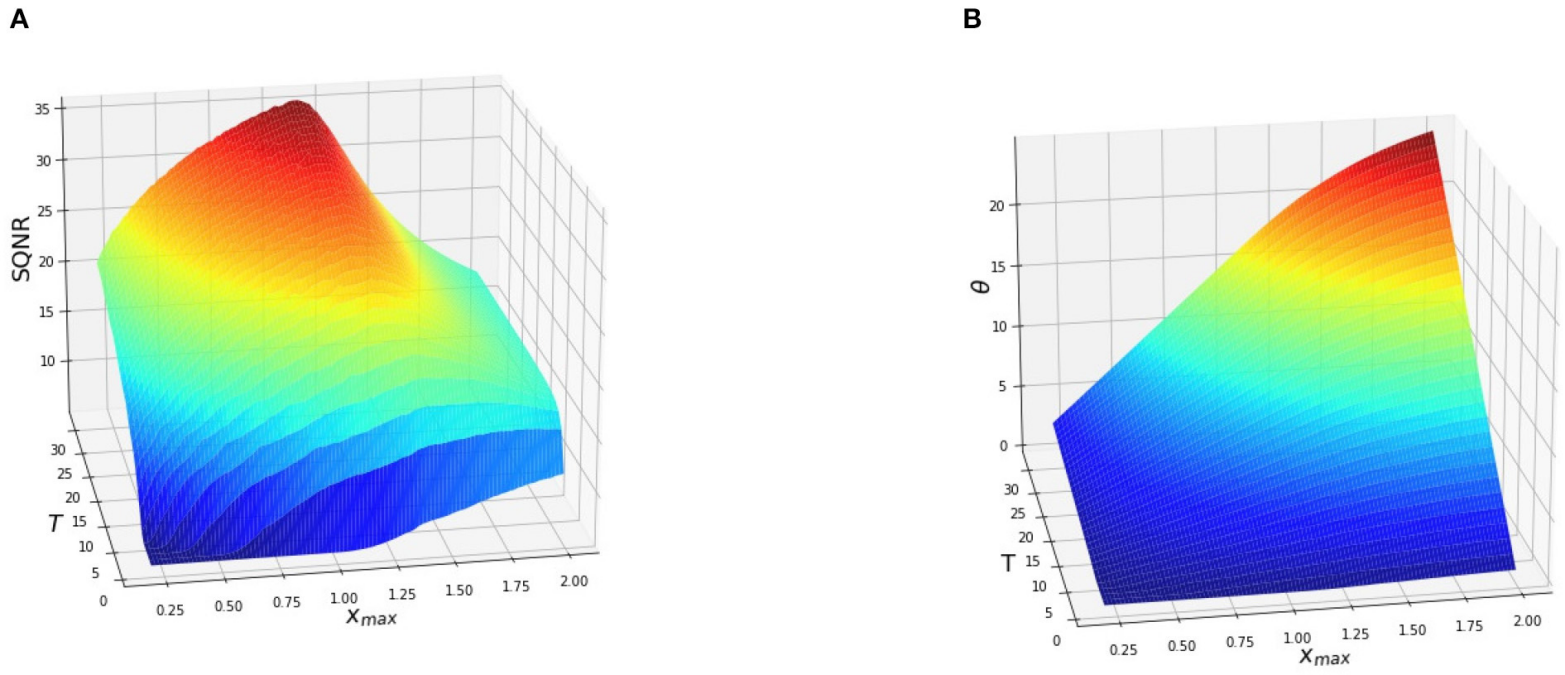
Average SQNR **(A)** and θ **(B)** of an IF neuron with soft reset as a function of the input range *x*_*max*_ and the integration time *T*. The pixels of the test images have been rescaled to the range [0, *x*_*max*_] where 0.25 ≤ *x*_*max*_ ≤ 2. Here, *V*_*th*_ = 1. and *b* = 0., therefore the quantization range spans the interval [0, 1.]. The SQNR decreases when *x*_*max*_ moves away from *V*_*th*_.

Here, *n* and *m* represent the height and the width of the input image. Finally, we report the average value of SQNR and θ on the test set. The average value of SQNR and θ as a function of *T* and *x*_*max*_ are shown in Figure 2.

As it can be observed from Figure 2 the SQNR increases using a longer integration time, that is increasing *T*, independently of the range of the input. Each additional timestep brings a new quantization step, thus leading to a finer discretization of the input and a higher SNQR. However, when *x*_*max*_ ≤ *V*_*th*_ the SQNR decreases since the number of quantization intervals available to encode the signal is lower than the total number of intervals that the encoder could theoretically provide. As an example, when *x*_*max*_ = 0.5 the input occupies only half the quantization range available. Therefore, only 50% of the quantization intervals are effectively used for the encoding which leads to a strong decrease in the SQNR. In the same way, when *x*_*max*_≥*V*_*th*_ the SQNR decreases since a part of the input signal (*x*_*max*_−*V*_*th*_) falls in the saturation zone of the quantizer. The input samples that fall in this interval will be converted to the same output regardless of their respective values. The sparsity, that is the number of spikes generated by each neuron are also affected by the choice of the parameters [*V*_*th*_, *T, x*_*max*_] as it can be observed from Figure 2. Moreover, we can see that it is not always useful to generate more spikes to increase the SQNR. For example, θ increases when *x*_*max*_≥*V*_*th*_ but the performance of the quantizer is lower. At the opposite, when *x*_*max*_ ≤ *V*_*th*_, only a fraction of the quantization steps allowed by latency *T* are used thus leading to a lower SQNR.

A solution to the problem related to the width of the quantization interval is to modify *V*_*th*_ to match the range of the input (*x*_*max*_). In this way, all the quantization intervals fall inside the input range, thus maximizing the SQNR without generating more spikes than necessary. The proposed quantization-aware training strategy is discussed in the next section.

### 3.2. Improving compression through trainable quantization and surrogate gradient learning

#### 3.2.1. Threshold scaling and gain compensation

**Figure 3:**
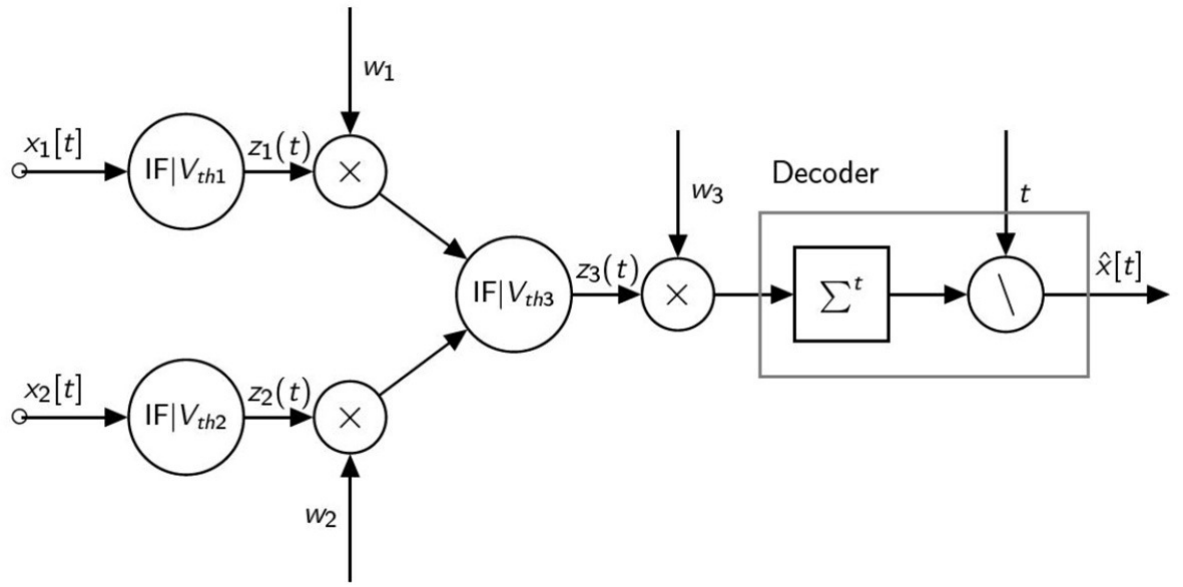
Minimal network model.

In this section, we provide a numerical example to explain how the firing thresholds can be adapted in a network of spiking neurons. Our example is based on the network shown in Figure 3 and composed of three identical spiking neurons arranged in two layers and followed by a rate decoder.

We consider two constant inputs with value (*x*_1_, *x*_2_) = (1.5, 1.2) and the following weights (*w*_1_, *w*_2_, *w*_3_) = (2, 3, 1). The biases are set to *b* = 0. to shorten the calculations. The thresholds are set to (*V*_*th*1_, *V*_*th*2_, *V*_*th*3_) = (1, 1, 1) and we also use *T* = 8 timesteps.

According to Equations (1) and (2), we can compute the output of each neuron and the decoded value $\hat{x}$ as follows:


(5) $$\begin{array}{ll} \overset{T}{\underset{t=1}{\sum }}z_{1}\left(t\right) & ={\left\lfloor \frac{\left(x_{1}+b\right)T}{V_{th1}}\right\rfloor }_{T}={\left\lfloor \frac{1.5\times 8}{1}\right\rfloor }_{T}=8\text{ spikes} \end{array}$$

(6) $$\begin{array}{ll} \overset{T}{\underset{t=1}{\sum }}z_{2}\left(t\right) & ={\left\lfloor \frac{\left(x_{2}+b\right)T}{V_{th2}}\right\rfloor }_{T}={\left\lfloor \frac{1.2\times 8}{1}\right\rfloor }_{T}=8\text{ spikes} \end{array}$$

(7) $$\begin{array}{l} \begin{array}{ll} \sum_{t=1}^{T}z_{3}(t) & ={\left\lfloor \frac{{\sum^{\text{​}}}_{t=1}^{T}(w_{1}V_{th1}z_{1}(t)+w_{2}V_{th2}z_{2}(t))+bT}{V_{th3}}\right\rfloor }_{T} \end{array} \end{array}$$

(8) $$\begin{array}{l} ={\left\lfloor \frac{2\times 8+3\times 8}{1}\right\rfloor }_{T}=8\text{ spikes} \end{array}$$

(9) $$\begin{array}{ll} \hat{x} & =\frac{8}{8}\times w_{3}=1.0 \end{array}$$

Using formal instead of spiking neurons the previous operation would have produced the following result:


(10) $$\begin{array}{l} x_{f}=\left(x_{1}w_{1}+x_{2}w_{2}\right)w_{3}=\left(1.5\times 2+1.2\times 3\times 1\right)=6.6 \end{array}$$

Using the previous results we can compute the difference between the spiking and formal outputs as follows:


(11) $$\begin{array}{l} \Delta_{s}=|x_{f}-\hat{x}|=6.6-1.0=5.6 \end{array}$$

This error Δ_*s*_ is caused by the mismatch at the input, since *x*_1_>*V*_*th*1_ and *x*_2_>*V*_*th*2_. Moreover, the output neuron is also mismatched since the sum of the activations is greater than *V*_*th*3_.

To correct the mismatch we can set (*V*_*th*1_, *V*_*th*2_, *V*_*th*3_) = (1.5, 1.5, 7), that is to the maximum value of the input of each synapse. However, by varying the thresholds a gain factor is introduced in the operation carried out by the neuron. Let us consider a spiking neuron with *V*_*th*_ = 1, its firing rate will be equal to 1, its maximum value, when its input is also equal to 1. If we decrease the threshold to *V*_*th*_ = 0.5, then the neuron outputs its maximum firing rate when the input is equal to 0.5. In this case the neuron is introducing a gain of 1/*V*_*th*_ = 2. To compensate the gain introduced by the neuron at layer *l* we can simply multiply the weights of the post-synaptic neurons at layer *l*+1 by the coefficient 1/*V*_*t*_*h*__*l*__, that is the threshold of the neurons at layer *l*.

Following the previous example, and using the new threshold values and the gain compensation, we can compute the decoded value $\hat{x}$ as follows:


(12) $$\begin{array}{ll} \overset{T}{\underset{t=1}{\sum }}z_{1}\left(t\right) & ={\left\lfloor \frac{\left(x_{1}+b\right)T}{V_{th1}}\right\rfloor }_{T}={\left\lfloor \frac{1.5\times 8}{1.5}\right\rfloor }_{T}=8\text{ spikes} \end{array}$$

(13) $$\begin{array}{ll} \overset{T}{\underset{t=1}{\sum }}z_{2}\left(t\right) & ={\left\lfloor \frac{\left(x_{2}+b\right)T}{V_{th2}}\right\rfloor }_{T}={\left\lfloor \frac{1.2\times 8}{1.5}\right\rfloor }_{T}=6\text{ spikes} \end{array}$$

(14) $$\begin{array}{l} \begin{array}{ll} \sum_{t=1}^{T}z_{3}(t) & ={\left\lfloor \frac{{\sum^{\text{​}}}_{t=1}^{T}(w_{1}V_{th1}z_{1}(t)+w_{2}V_{th2}z_{2}(t))+bT}{V_{th3}}\right\rfloor }_{T} \end{array} \end{array}$$

(15) $$\begin{array}{l} ={\left\lfloor \frac{3\times 1.5\times 8+2\times 1.5\times 6}{7}\right\rfloor }_{T}=7\text{ spikes} \end{array}$$

(16) $$\begin{array}{ll} \hat{x} & =\frac{7}{8}\times w_{3}\times V_{th3}=\frac{7}{8}\times 7=6.125 \end{array}$$

Following this approach, the quantization error is now equal to:


(17) $$\begin{array}{l} \Delta_{s}=|{\hat{x}}_{f}-\hat{x}|=6.6-6.125=0.475 \end{array}$$

It corresponds to more than a tenfold decrease in the error compared to the previous example, with less spikes (21 compared to 24 in the mismatched configuration).

The previous example allowed us to introduce the gains that can be obtained by setting the neuron parameters to minimize the input mismatch. However, *V*_*th*_ was set manually by comparing quantized and full-precision operations. Moreover, we set *V*_*th*_ to the maximum value of the input synapses. Better choices and further gains can be obtained by learning *V*_*th*_ rather than tuning it layer-by-layer using a post-training procedure.

#### 3.2.2. Learning the spiking neuron parameters - the ATIF neuron model

The surrogate gradient method (Neftci et al., 2019) used in SNNs allows computing the derivative of the Heaviside step function of a spiking neuron during error back-propagation, thus making the full spiking network trainable using BPTT and standard deep learning frameworks. Specifically the non-differentiable Heaviside step function Θ(*x*) is replaced during back-propagation with Θ′(*x*) = σ′(*x*). Where σ(*x*) is an approximation of the step function, typically a sigmoid, used in the following experimentation, or any other form of smooth saturating function.

The upstream gradient of the spiking neuron, $\frac{\partial L}{\partial z}$, can be propagated to *V*_*th*_ thanks to the surrogate function:


(18) $$\begin{array}{l} \frac{\partial L}{\partial V_{th}}=-\frac{\partial L}{\partial z}\cdot \sigma^{\prime }\left(q\right) \end{array}$$

Where *q* denotes the membrane potential of the neuron. It is thus possible to optimize both the weights and biases of the network and the *V*_*th*_ of the spiking neurons during gradient descent. In consequence, both the task loss and the quantization error can be jointly optimized. This spiking neuron model, called ATIF, has been introduced in Castagnetti et al. (2023b) and tested on image and sound classification tasks. In the following sections, we experimentally evaluate the ATIF neuron model for the task of image denoising. We then provide a comparison between the ATIF-based SNN networks, quantized-ANN and other SNN-based image denoising networks based on different spiking neuron models and coding mechanisms.

## 4. Experiments and results

### 4.1. Experimental setting

We train the model proposed in Zhang et al. (2017), called DnCNN, for the task of denoising with known specific noise level. The network is composed of 17 convolutional layers each with 64 filters. The input are gray-scale images, with pixel values normalized in the interval [0 − 1]. We use the same dataset as Zhang et al. (2017) for training and testing. The training set is composed of 400 images of size 180 × 180. The training images are first cropped into patches of size 40 × 40 and noise is added to each patch. We evaluate the models on two different noise types: additive Gaussian and multiplicative Speckle noise. Then, the patches are flowed into the network. We test three different noise levels with standard deviations σ = [15, 25, 50] respectively. The higher the σ the harder the denoising task. For training we follow the residual learning formulation proposed in the original paper. The network *R*(·) is fed with a noisy image *y* = *x*+*v*, where *x* is the clean image and *v* is the noise and must output the residual mapping *R*(*y*)≈*v*. We use the Peak-Signal-to-Noise-Ratio (PSNR) between the clean image *x* and *x*_*d*_ = *y*−*R*(*y*) to measure the denoising performance of the network. The higher the PSNR the better.

**Figure 4:**
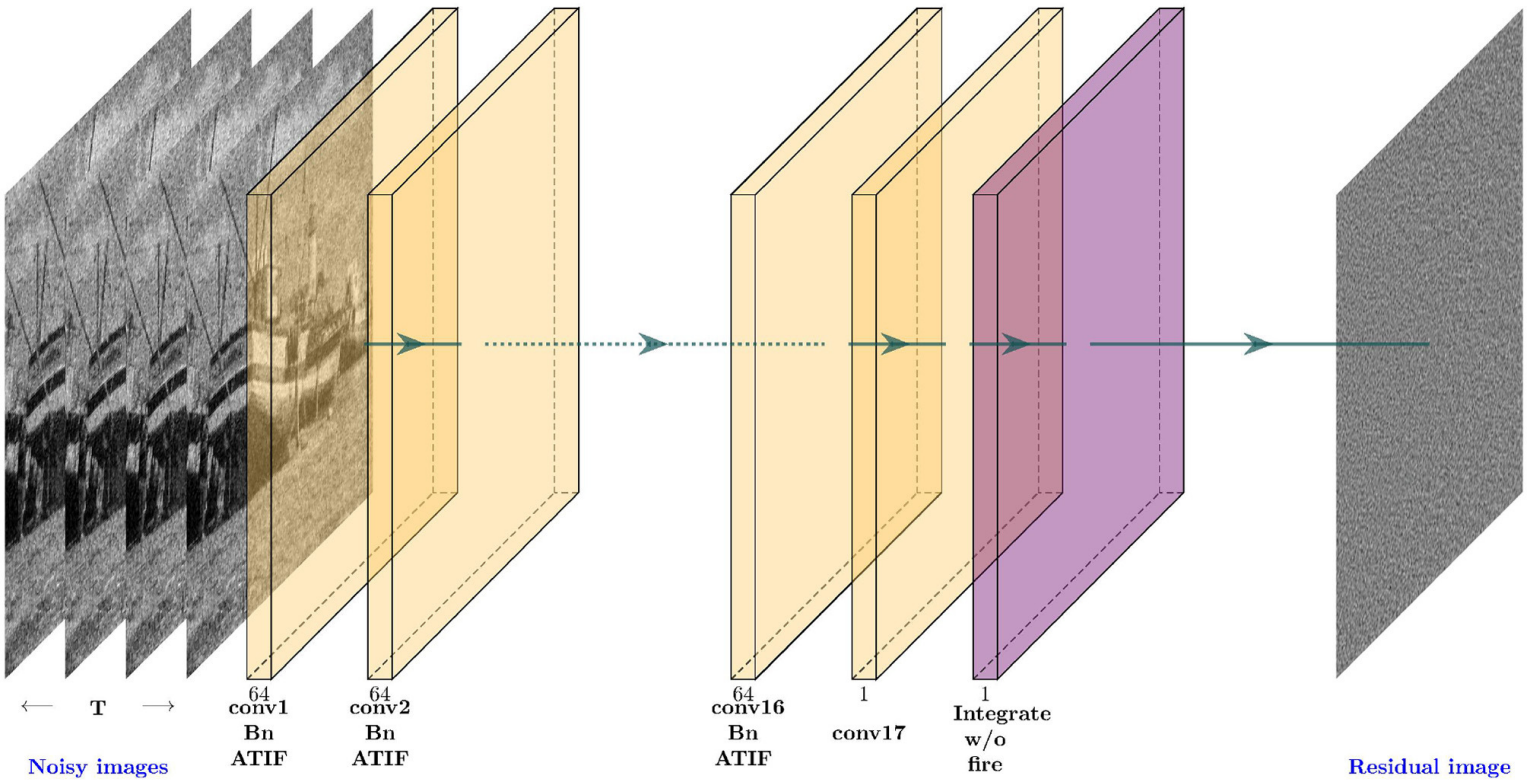
The architecture of DnSNN as well as the encoding (image to spike) and decoding (spike to image) processes.

For the spiking denoiser shown in Figure 4, that we call DnSNN, we replace the *ReLU* activation functions with the spiking ATIF neurons described in Section 3.2. The image to spike conversion process is carried out by the first layer of the network. The noisy image *y* is forwarded *T* times at the input of the network. Then the spiking neurons that follow the first convolutional layer (conv1) converts their membrane potentials into a train of spikes. The decoding process is carried out at the last layer, where the outputs of the last convolution (conv17) is collected by a read-out layer composed of integrate without fire neurons. These neurons accumulate the output of the conv17 layer at each timestep. The residual image *v* is then obtained by reading-out the membrane potentials of these neurons at the last timestep. Finally, after the last timestep and before feeding a new image to the network, the membrane potential of each spiking neuron is hard reset between each image. We use PyTorch and the SpikingJelly framework (Fang et al., 2020) for simulating DnSNN. Both networks are trained using Adam optimizer with a learning rate $l_{r}=10^{-3}$. The learning rate is exponentially decayed with a factor of 0.1 each 10 epochs. Finally, the networks are trained for 30 epochs. For fair comparison with previous works we report the performance for the same test sets used in Zhang et al. (2017). These datasets, composed of natural images, are widely used for the evaluation of gaussian denoising methods. The first dataset contains 68 natural images from the Berkeley segmentation dataset (BSD68) and the second one (Set12) contains 12 images. None of the test images are included in the train dataset.

### 4.2. Quantitative and qualitative image denoising evaluation

#### 4.2.1. Gaussian denoising

In this section, an Additive White Gaussian Noise (AWGN) with standard deviation σ is considered as noise model. The noisy image is defined by the following degradation model:


(19) $$\begin{array}{l} y=x+v \end{array}$$

Where *x* is the clean image and *v*~*N*(0, σ) is the gaussian distributed additive noise. We first compare DnSNN with a full-precision implementation of DnCNN where all the network parameters and neurons activations are encoded using a 32 bits floating point (FP) representation. Both DnCNN and DnSNN have the same architecture and number of parameters (556K trainable parameters). The experimental results for both BSD68 and Set12 are shown in Table 1.

**Table 1:** The average PSNR (dB) results of DnCNN (Zhang et al., 2017), FC-AIDE (Cha and Moon, 2019), FOC-Net (Jia et al., 2019), and DnSNN on the Set12 and BSD68 datasets for three different noise levels.

	**FC-AIDE**	**FOC-Net**	**DnCNN**	**DnSNN**		**DnCNN vs. DnSNN**
**Set12**						
	**PSNR [dB]**	**PSNR [dB]**	**PSNR [dB]**	**PSNR [dB]**	θ/*T*	Δ_*PSNR*_ **[dB]**
σ = 15	32.99	33.07	32.859	32.681	0.282	0.178
σ = 25	30.57	30.73	30.436	30.186	0.295	0.25
σ = 50	27.42	27.68	27.178	26.790	0.31	0.388
**BSD68**						
σ = 15	31.78	31.83	31.73	31.593	0.285	0.137
σ = 25	29.31	29.38	29.23	29.025	0.293	0.205
σ = 50	26.38	26.50	26.23	25.94	0.31	0.29

The results for DnSNN are given for *T* = 15 timesteps. The normalized sparsity of DnSNN is also reported.

**Figure 5:**
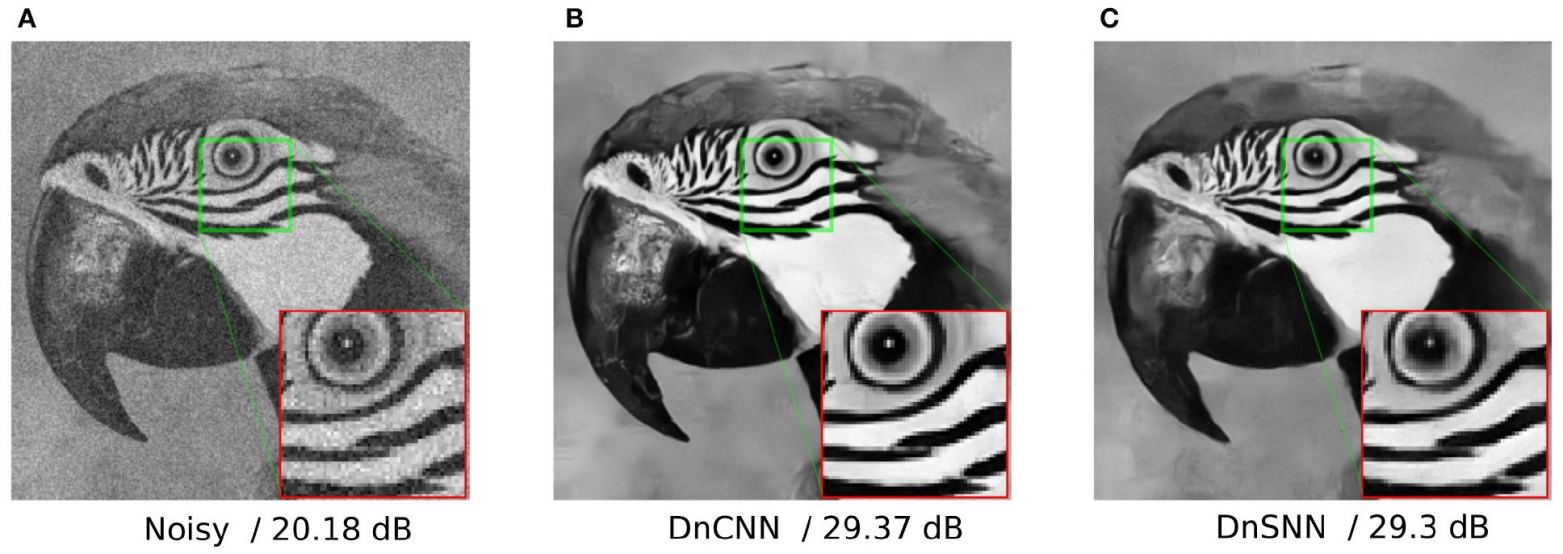
Gaussian denoising results of the image *parrot* with noise level 25. The result for DnSNN is given for *T* = 15 timesteps. **(A)** Noisy/20.18 dB. **(B)** DnCNN/29.37 dB. **(C)** DnSNN/29.3 dB.

As it can be observed, DnSNN provides competitive results in terms of PSNR compared to a non-quantized DnCNN network. As an example, we achieve an average PSNR of 31.593 dB on the BSD68 dataset with σ = 15, which is only 0.137 dB below the PSNR provided by the DnCNN. The performance gap (Δ_*PSNR*_) increases for higher levels of input noise, but always stays below 0.4 dB in all the considered scenarios. As discussed in Section 3.1 spiking neurons introduce a quantization noise in their output because of the discretization introduced by the spikes. At the opposite, the quantization noise is absent from the FP implementation of DnCNN, thus explaining the performance gap. However, as our results suggest, by jointly minimizing the SQNR of neurons, i.e., learning *V*_*th*_, and the task loss we can minimize the performance degradation while keeping the inference latency, i.e., *T*, low. Moreover, these results are achieved with a significant level of sparsity since DnSNN generates 0.3 spikes/neuron/timestep on average. As it will be discussed later, the sparsity besides the latency is another key parameter that must be minimized to get energy gains. Finally, the denoising results of both DnCNN and DnSNN for a specific image are shown in Figure 5. As it can be observed, the denoised image generated by DnSNN matches the quality of the DnCNN output: edges and details are recovered and the effect of the quantization noise does not generate artifacts in the smooth regions.

#### 4.2.2. Speckle denoising

In this section, both DnCNN and DnSNN are evaluated using a multiplicative noise model called *Speckle* noise. The degradation model is defined as follows:


(20) $$\begin{array}{l} y=x+x\times v \end{array}$$

Where the multiplicative noise factor is gaussian distributed *v*~*N*(0, σ). Multiplicative Speckle noise is common in medical images obtained using ultrasound and optical coherence tomography (Nao and Wang, 2022).

We report the experimental results on Table 2.

**Table 2:** The average PSNR (dB) results of DnCNN (Zhang et al., 2017) and DnSNN on the Set12 dataset for Speckle denoising.

	**DnCNN**	**DnSNN**		**Δ_*PSNR*_ [dB]**
	**PSNR [dB]**	**PSNR [dB]**	**θ/*T***	
σ = 15	29.63	29.06	0.329	0.57
σ = 25	28.51	27.86	0.324	0.65
σ = 35	27.83	27.12	0.325	0.71

The results for DnSNN are given for *T* = 8 timesteps. The normalized sparsity of DnSNN is also reported.

**Figure 6:**
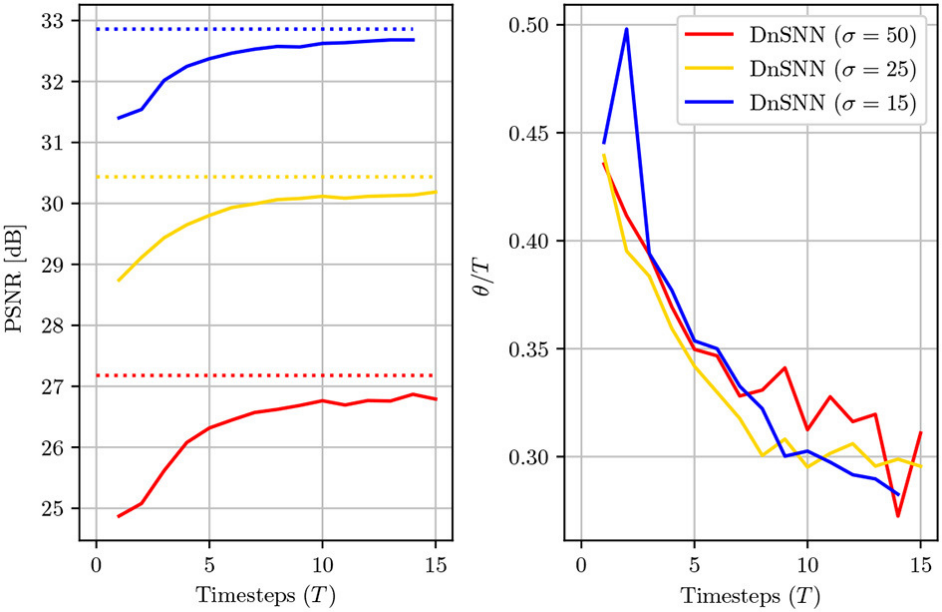
The average PSNR (dB) and the normalized sparsity (θ/*T*) of DnSNN on the Set12 dataset for noise levels σ = [15, 25, 50] and *T*∈[1, 15] timesteps. The PSNR levels for DnCNN are also shown with dotted lines for each noise level.

For Speckle denoising we observe that the performance of both networks are lower compared to the case of Gaussian denoising. This is explained by the increasing complexity of the noise model which has a multiplicative relationship with the original signal. As it can be observed, the performance gap in terms of PSNR between DnSNN and DnCNN is slightly higher compared to Gaussian denoising for the same number of timesteps. As an example, we can observe from Figure 6 that for Gaussian denoising with σ = 25 and *T* = 8 timesteps, the Δ_*PSNR*_ = 0.374 dB. For Speckle noise and the same configuration of noise value and timesteps the Δ_*PSNR*_ = 0.65 dB, a loss of 0.27 dB compared to the gaussian case. However, we can observe that the noise type does not significantly impact the sparsity of the network. Again, as a comparison we obtain a sparsity θ = 0.3 for Gaussian denoising and θ = 0.324 for Speckle denosing, both for *T* = 8 timesteps. These results show that the proposed method performs well with different types of noise. However, due to the increased complexity of the Speckle noise, more timesteps are needed to match the performance of the DnCNN network. In the following sections we only consider Gaussian denoising.

### 4.3. Latency/performance trade-off

The temporal dimension of SNNs allows to trade neurons activation precision with latency, thus giving access to a trade-off in terms of latency and performance. We study the effect of the inference latency on the DnSNN performance on the Set12 dataset for different noise levels. The PSNR and sparsity are shown in Figure 6 as a function of the inference latency.

**Figure 7:**
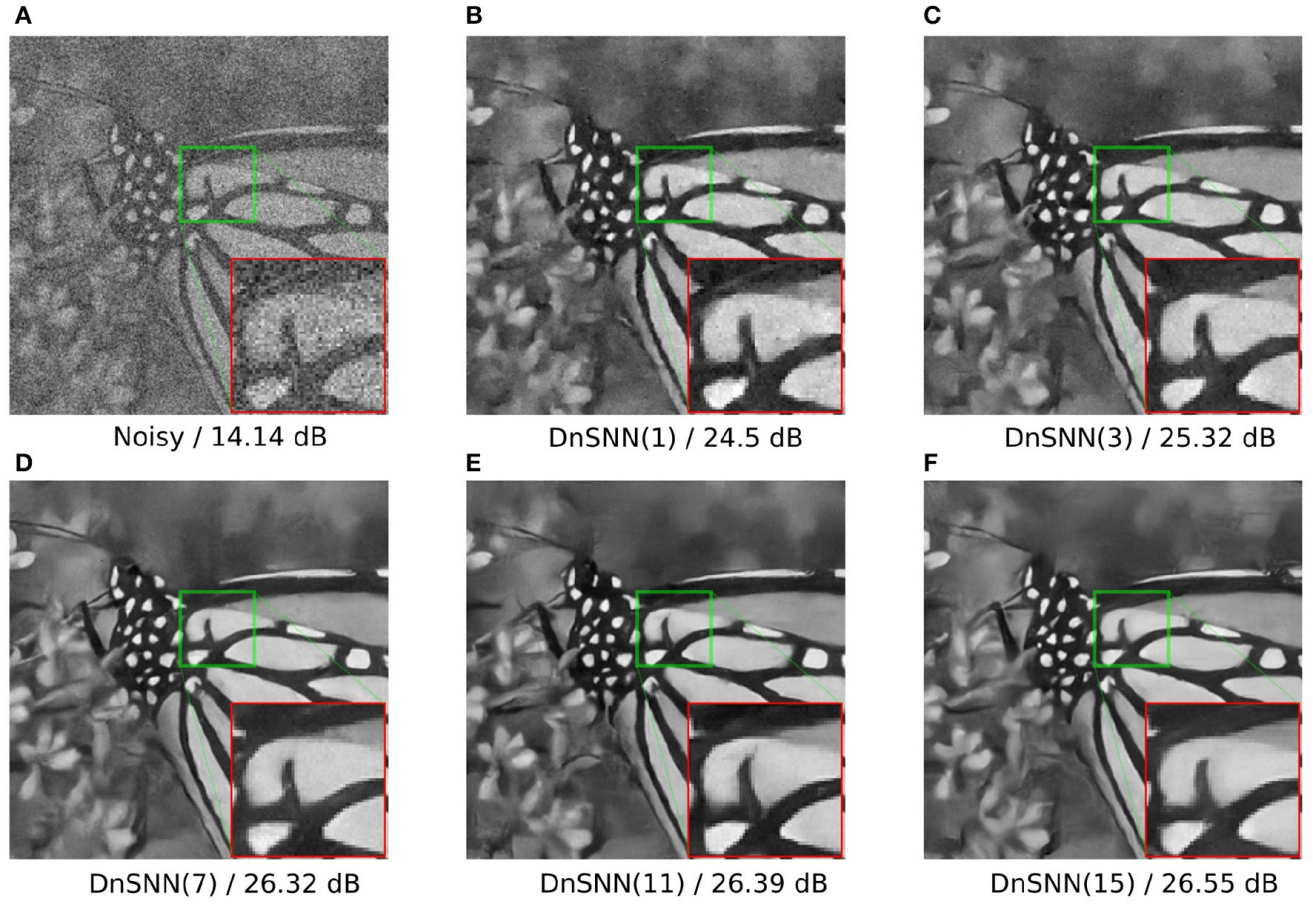
DnSNN Gaussian denoising results of the image *butterfly* with noise level 50 for different number of timesteps. The latency is shown in parenthesis. **(A)** Noisy/14.14 dB. **(B)** DnSNN(1)/24.5 dB. **(C)** DnSNN(3)/25.32 dB. **(D)** DnSNN(7)/26.32 dB. **(E)** DnSNN(11)/26.39 dB. **(F)** DnSNN(15)/26.55 dB.

As expected, by reducing the latency the performance of the DnSNN drops compared to the full-precision DnCNN. By lowering the latency we indeed decrease the number of quantization intervals provided by each spiking neuron. In consequence, more quantization noise is present at the neurons outputs leading to a decrease in the performance. Moreover, the normalized sparsity, that is the average number of spikes generated at each timestep decreases for longer inference latencies, suggesting that the spike generation process does not scale linearly with the latency. However, to measure the total activity generated by DnSNN one has to consider the total amount of spikes generated during the whole inference process. For example, we can observe that when *T* = 1 each neuron generates on average 0.45 spikes per inference. At the opposite when *T* = 15 each neuron fires 0.3 × 15 = 4.5 spikes per inference, thus generating a greater activity and potentially reducing the expected energy gains. Finally, we show in Figure 7 the denoising results for the DnSNN networks for different inference latencies. As it can be observed, several artifacts are visible in the smooth regions of the images generated using short latency inferences, e.g., *T* = 1 and *T* = 3. Increasing the latency smoothens the quantization artifacts that are almost invisible when *T* = 15.

### 4.4. Comparison with multi-resolution denoising networks

In the previous sections, we have assessed the performance of the ATIF neuron for the DnCNN architecture. DnCNN is a particular form of autoencoder which does not modify the dimensions over the layers. Here, we analyze the performance of the ATIF neuron model for the particular case of a multi-resolution denoising convolutional autoencoders, that we call CAE-F and CAE-S for the formal and spiking version respectively. The CAE denoiser is composed of 8 convolutional layers, each composed of 64 filters. The first and the last convolutional layers do not modify the input dimensions as in DnCNN. At the opposite, the intermediate layers first down-sample then up-sample the inputs therefore generating a bottleneck in the middle of the network. Specifically, the second, third and fourth layers down-sample the inputs, each by a factor of two. This is achieved using normal convolutions with a stride of two. The following layers up-sample their respective inputs in order to obtain, at the output of the seventh layer, the same dimension of the input image. The up-sampling operation is achieved using fractionally-strided convolutions with a stride of two. Each convolutional layer is followed by a *ReLU* activation function, in the CAE-F configuration, and by an ATIF spiking neuron, in the CAE-S configuration. The experimental results of both networks for two noise levels are shown in Table 3. As we can observe, due to the reduced depth of the CAE networks the denoising performance are lower compared to both original DnCNN and DnSNN. However, the performance difference between CAE-F and CAE-S, measured by Δ_*PSNR*_, is similar to that previously observed for DnCNN for the same noise levels. As shown in Table 1, for a noise level σ = 25, the performance gap between DnCNN and DnSNN is 0.25 dB on the Set12 dataset. On the same dataset and for the same noise level the performance gap between CAE-F and CAE-S is 0.21 dB. The same trend can also be observed for the BSD68 dataset and with different noise levels on the input images. These results suggest that the proposed spiking neuron model is able to minimize the quantization error, therefore limiting the performance gap between CNN and SNN, even for multi-resolution networks with dimensionality reduction over the layers.

**Table 3:** The average PSNR (dB) results of CAE-F and CAE-S on the Set12 and BSD68 datasets for two different noise levels.

	**CAE-F**	**CAE-S**		**CAE-F vs. CAE-S**
**Set12**				
	**PSNR [dB]**	**PSNR [dB]**	θ/*T*	Δ_*PSNR*_ **[dB]**
σ = 15	31.32	31.24	0.34	0.08
σ = 25	28.45	28.24	0.35	0.21
**BSD68**				
σ = 15	30.51	30.48	0.35	0.03
σ = 25	27.65	27.53	0.37	0.12

The results for CAE-S are given for *T* = 8 timesteps. The normalized sparsity of CAE-S is also reported.

### 4.5. Comparison with other SNN-based image denoising methods

**Figure 8:**
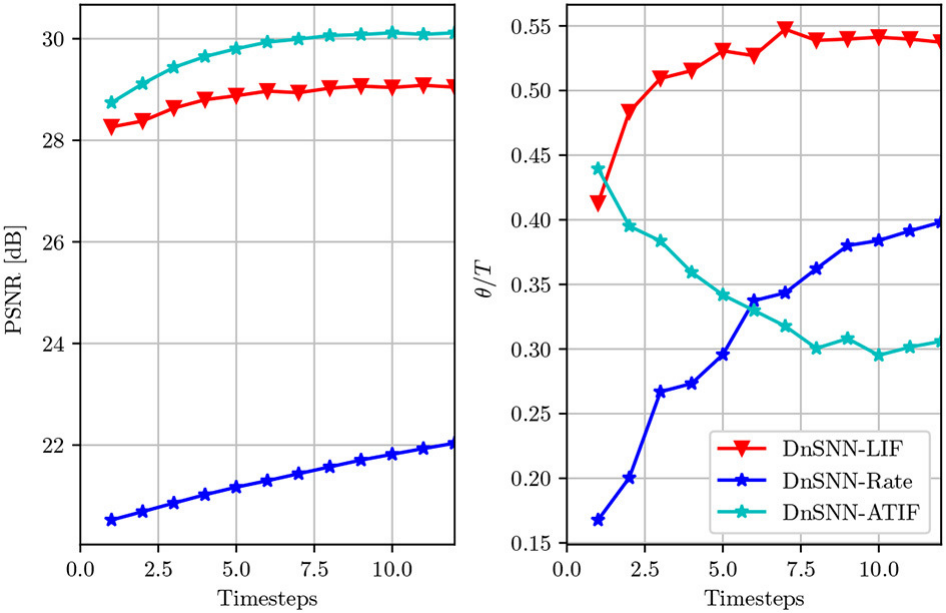
The average PSNR (dB) and the normalized sparsity (θ/*T*) of DnSNN-ATIF, DnSNN-LIF, and DnSNN-Rate on the Set12 dataset for noise levels σ = 25 and *T*∈[1, 12] timesteps.

To highlight the benefit of the ATIF spiking neuron model discussed in Section 3.2, we compare the previous results obtained with the SNN-based image denoising solutions introduced in Castagnetti et al. (2023a). Here, two different SNNs are developed for the task of image denoising and tested on the same benchmark dataset. The first network, that we call DnSNN-LIF is similar to our DnSNN, but the *ReLU* activation functions are replaced with Leaky Integrate and Fire (LIF) neurons. In this implementation the spiking neuron parameters (e.g., *V*_*th*_, τ) are fixed at the design stage and only the weights and the biases of the neural networks are learned during training. The second network, called DnSNN-Rate shares a similar architecture with DnSNN-LIF, but uses rate-coded images as input. In the rate coding scheme each pixel with intensity *x*_*i*_ is first normalized between [0, 1] and then converted into a spike train with rate *r*_*i*_. In this coding scheme, each spike train is an independent realization of a Poisson process with rate *r*_*i*_. The PSNR and the sparsity as a function of the inference latency are shown in Figure 8.

As it can be observed, the higher the inference latency *T* for each DnSNN, the better the PSNR. However, the PSNR is on average 1 dB lower for DnSNN-LIF compared to DnSNN with ATIF neurons. As this result suggests, LIF neurons introduce more quantization noise compared to the proposed model. In fact, as shown in Castagnetti et al. (2023a), the LIF neurons implement a non-uniform quantization scheme, where the size of each quantization step depends on the leakage current. As a result, the non-uniform quantization scheme that emerges from the LIF neuron generates more distortion, thus a lower SQNR compared to the ATIF model. Moreover, the parameters of the spiking neurons in DnSNN-LIF cannot be modified during training and the quantization noise cannot be further reduced. On the other hand, in the rate conversion scheme the PSNR increases with a logarithmic shape as a function of *T*. However, due to the stochastic nature of the generation process, a significant amount of information is lost during the conversion of pixels into spike trains. In consequence, DnSNN-Rate produces a PSNR which is almost 8 dB lower compared to the other SNNs. We can also observe, that the specific quantization scheme of the LIF neuron generates more spikes compared to the ATIF model. For example, for *T* = 10, each LIF neuron generates on average 0.54 × 10 = 5.4 spikes per inference, while the ATIF neuron only emits 0.3 × 10 = 3, so almost 80% less activity. The same observation holds for the rate coding scheme, where spikes are generated at an almost linear rate.

### 4.6. Comparison with quantized ANN

In the last sections, we have compared the performance of DnSNN with an FP implementation of DnCNN. As previously discussed, using an FP representation for the neurons activations does not introduce any measurable quantization noise in the network. So, to make a fair comparison, we train DnCNN using a Quantization-Aware-Training technique called LSQ (Esser et al., 2020). In the following, we call this network q-DnCNN. It is worth noticing that only the DnCNN neurons activations are quantized. The weights, biases and the other networks parameters are still coded using FP representation in both q-DnCNN and DnSNN. In q-DnCNN each neuron's *ReLU* activation is replaced with the discretized version of the same function. The number of quantization levels is fixed before training, specifying the number of bits (bitwidth) used to map real numbers to a set of discrete values. As an example, using a 4-bit quantization the outputs of the neurons are mapped to 2^4^ = 16 different quantization levels. Using low-precision representations one can therefore trade performance with computational and memory costs, similarly to the latency-performance trade-off offered by SNNs. Moreover, LSQ provides a method to learn the quantization step-size (the width of a quantization bin) during training. By minimizing the SQNR, LSQ provides state of the art performance on complex image classification tasks even for very low-precision networks, e.g., 2-bit quantization (Esser et al., 2020). LSQ is therefore similar to the trainable quantization method described in Section 3.2 for spiking neurons. The spiking neuron model described in Section 3.1, provides *T*+1 quantization intervals for an inference latency *T* (Castagnetti et al., 2023b), we therefore expect similar results when quantizing the DnCNN activations using the following bitwidth:


(21) $$\begin{array}{l} b=\log_{2}\left(T+1\right) \end{array}$$

The experimental results are shown in Table 4. As it can be seen, DnSNN and q-DnCNN provide almost the same PSNR when the neurons activations quantization follows the relation given in Equation (21). In conclusion, DnSNN can reach the performance of a full-precision network like DnCNN and match the performance of the quantized version of the same network, q-DnCNN. Even if these networks perform similarly, the mechanisms underlying their operations are quite different: SNNs use multiple time-steps and sparse synaptic operations to perform their task, while ANNs use single time-step dense computations. In the following section, we explore the performance-energy trade-off that both networks offer in the context of the denoising task.

**Table 4:** The average PSNR (dB) of a quantized DnCNN (q-DnCNN) and DnSNN on the Set12 dataset for a σ = 25 noise level.

**q-DnCNN**		**DnSNN**		**Δ_*PSNR*_ [dB]**
**Bitwidth (*b*)**	**PSNR [dB]**	***T***	**PSNR [dB]**	
1	28.25	1	28.74	0.49
3	30.04	7	29.99	0.05
4	30.23	15	30.18	0.05

Each configuration of bitwidth and *T* leads to the same number of quantization intervals in both networks.

### 4.7. Energy efficiency

The metric introduced in Lemaire et al. (2022) is used to estimate the energy consumption of both DnCNN and DnSNN. This metric provides an hardware-independent way of estimating the energy consumption by taking into account the synaptic operations, memory accesses, and element addressing. The model takes into account the network architecture, the number of parameters and activations as well as the sparsity for the SNN. It outputs the number of synaptic operations, the Read/Write memory accesses and the operations needed to address the memory. The energy is then computed by multiplying the energy cost of each operation by its number of occurrences. To compute the energy per operation we use the estimation provided by Jouppi et al. (2021) which refers to a 45 nm process. Moreover, to estimate the memory accesses the metric makes the assumption that each layer of both the ANN and the SNN has its own local (non-shared) memory. The memory, assumed to be local SRAM, stores the parameters (weights, biases) and also serves as a buffer for the activations of each layer. In the case of SNN the memory also stores the membrane potentials of the spiking neurons. An input image of size 256 × 256 is considered for all the following results.

#### 4.7.1. Latency/energy trade-off

**Figure 9:**
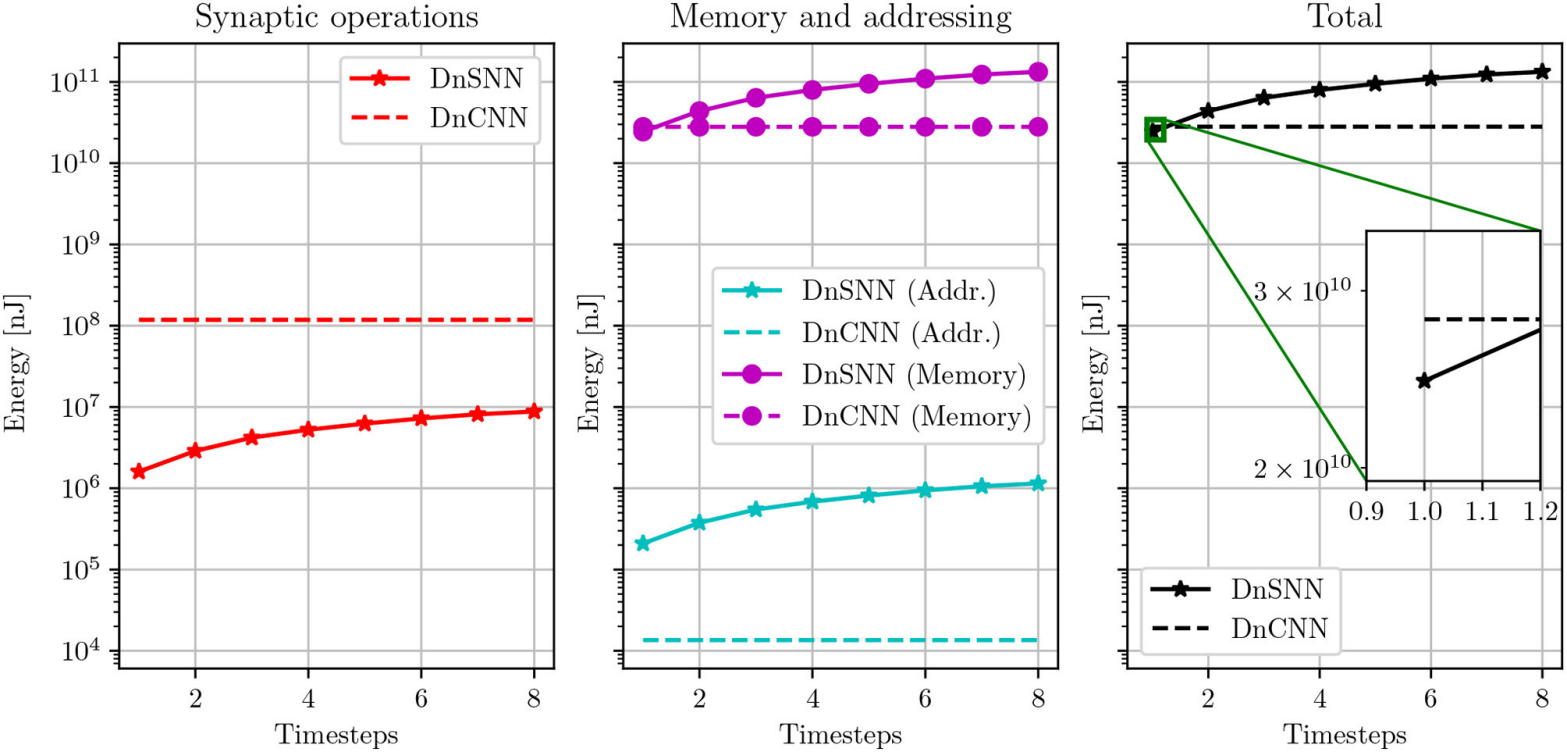
Energy consumption estimation of DnSNN for different inference latencies. The energy consumption of DnCNN, which is independent on the number of timesteps, is also reported. Energies are shown in log-scale.

Based on the results presented in Section 4.3, we discuss the latency-energy trade-off for DnSNN. Figure 9 shows the energy consumption of DnSNN as a function of the latency, *T*. As a reference, the energy consumption of DnCNN, which is independent of the parameter *T*, is also shown.

As it can be observed, the energy consumption of DnSNN increases for longer inference latencies. As discussed in Section 4.3, the longer the latency the higher the activity, that is the number of spikes generated during the inference process. As SNNs are event-driven, synaptic operations as well as the associated memory accesses are indeed triggered upon the reception of spikes. Therefore, the lower the total number of spikes the lower the energy consumption. It can also be observed that the total energy consumed by synaptic operations for DnSNN is one to two order of magnitude lower compared to DnCNN. This outcome was expected according to what is often reported in the SNN literature (Sengupta et al., 2019). For spiking neurons, each synaptic operations requires an addition of the weights into the membrane potential instead of the costly multiply-accumulate operation required by ANNs, thus explaining the energy reduction for these operations. However, we can observe that the energy consumption is completely dominated by the memory accesses. This fact has already been observed in previous works (Lemaire et al., 2022) for classification tasks using different neural network architectures. For the DnSNN architecture, the memory access cost is particularly high. This is due to the amount of memory accesses needed by this particular architecture. The neural architecture presented in Zhang et al. (2017) and used in this work, is a particular form of autoencoder where there is no data dimensionality reduction throughout the layers. This makes the memory requirement for storing the membrane potentials particularly high. We can observe that DnSNN consumes less energy than DnCNN only for *T* = 1 timesteps. Specifically DnSNN consumes 24.4·10^9^ nJ compared to 28.1·10^9^ nJ for DnCNN, a 15% reduction in energy consumption. However, for higher latency the energy consumption of DnSNN increases, making it less energy efficient compared to DnCNN. As an example, for *T* = 2 DnSNN consume almost 30% more energy than DnCNN.

The previous results suggests that to obtain an energy efficiency improvement for a given neural network architecture, one has to reduce not only the inference latency, but also the amount of spikes generated during the inference by the SNN network, so the sparsity. In this section, we analyze the energy efficiency by comparing DnCNN and DnSNN for a given network configuration, i.e., number of layers and parameters. In the next section, we study the impact of the network configuration in terms of number of parameters on the energy efficiency.

#### 4.7.2. Network size/energy trade-off

Here, we investigate the impact of the network size on the DnSNN energy efficiency, specifically we modify the original DnCNN architecture and increase the number of filters per layer. We train both DnCNN and DnSNN with the following number of filters per layer: [64, 96, 128, 160]. The base model described in the previous section has 64 filters per layer. The PSNR and the sparsity for each configuration are reported in Table 5 for a noise level of 25 and *T* = 2.

**Table 5:** The average PSNR (dB) results of DnSNN on the Set12 dataset for a noise level of 25 and four different configurations of network size.

**Nb. filters**	**q-DnCNN**	**DnSNN**	
	**PSNR [dB]**	**PSNR [dB]**	**θ/*T***
64	28.25	29.11	0.422
96	28.89	29.13	0.407
128	29.25	29.21	0.366
160	29.4	29.44	0.342

DnSNN is trained with *T* = 2 and q-DnCNN with *b* = 1.

**Figure 10:**
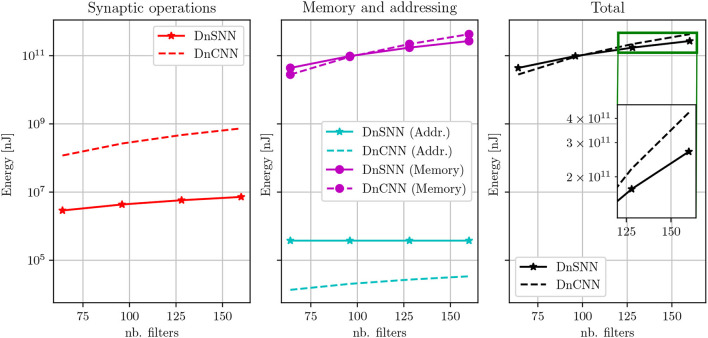
Energy consumption estimation of DnSNN and DnCNN for different network sizes. Both networks are trained with a noise level of 25. Here, *T* = 2 for DnSNN.

In Table 5, it can be seen that as the network size increases the performance and the sparsity of DnSNN improve. Moreover, by observing the energy consumption curves shown in Figure 10 we can notice that as the network size grows both the energy consumption of DnCNN and DnSNN increases.

However, for larger networks and thanks to the sparsity, the total amount of memory accesses is now lower for DnSNN than for DnCNN. As an example, with 128 filters per layer, DnSNN consumes 173·10^9^ nJ compared to the 220·10^9^ nJ of DnCNN, that is more than 20% energy gain. This gain becomes greater as the network grows, e.g., almost 40% in favor of DnSNN with 160 filters per layer.

In conclusion our results indicate that, unlike what is often assumed in the SNN research, the sole reduction of the energy consumption of synaptic operations provide an energy advantage for SNNs only for very small inference latencies, in our case only when *T* = 1. When memory costs dominate the energy budget, increasing the latency above few timesteps hinders the energy efficiency of SNNs. However, the effect of the sparsity is more pronounced for larger networks. The dense computation performed by the ANN requires accessing all the weights and activations in each layer, which causes an almost linear increase of the memory accesses. In SNNs, the memory accesses also grow but at a smaller pace thanks to the sparsity which results in an energy efficiency improvement.

## 5. Discussion and further improvements

In this work, we propose a method for training deep Spiking Neural Networks for the task of image denoising. To the best of our knowledge, this is the first work that addresses the problem of training deep SNNs for such a task. Image denoising is particularly difficult to deal with SNNs due to the challenge of predicting continuous values using the discretized representation of the information provided by the spikes.

Our results show that when taking into account the quantization noise introduced by the spiking neurons during training, it is then possible to separate noise from noisy observations with a performance level very close to a full-precision non-spiking ANN. In particular, our best results show that our proposed spiking denoising network, called DnSNN, achieves 31.593 dB of signal-to-noise-ratio on BSD68 dataset with noise level 15, which is only 0.137 dB below the performance of DnCNN. Similar results are also achieved for different noise levels and datasets, highlighting the robustness of DnSNN. We also report the latency and the sparsity of DnSNN and show that our best results are achieved with low-latency.

Finally, we provide an energy efficiency estimation of DnSNN, using an analytical model (Lemaire et al., 2022) that takes into account both the energy cost of synaptic operations and the memory access. Our results confirm that SNNs are indeed more energy efficient when considering only synaptic operations, but their energy efficiency is reduced when the energy consumption is dominated by the memory accesses. These results suggest that besides latency, another important parameter that has to be optimized to improve the energy efficiency is the sparsity of the network, or equivalently the number of spikes generated during an inference. Finally, we also show that with a given level of sparsity, imposed by the network architecture and the particular task, SNNs energy efficiency could increase when the network size increases. This fact suggests that larger neural network architectures could potentially benefit from the event-based and sparse computing mechanisms provided by the SNNs compared to smaller ones.

Our network is trained using surrogate gradient and back-propagation in the spike domain. This training strategy, even if effective has some drawbacks. Since time is taken into account during the training phase, inputs and gradients must be propagated for *T* timesteps during the forward and backward passes, making the overall process very costly in terms of computation and memory. Forward-propagation-through-time (FPTT) has recently been proposed (Kag and Saligrama, 2021) as an alternative to BPTT for training recurrent networks. This method can be used for training SNNs that are a particular class of recurrent networks. FPTT could help alleviate the memory cost of training, since the gradients are back-propagated at each timestep. Moreover, FPTT could also be useful to reduce the exploding and vanishing gradient problems that affect recurrent networks in general as well as SNNs.

Finally, in our work we did not consider the problem of quantizing the network parameters like the weights, biases and membrane potentials. However, we believe that this is an important subject that has to addressed in the future, notably to reduce the amount of memory needed in SNNs to store the membrane potentials of the spiking neurons.

## Data availability statement

Publicly available datasets were analyzed in this study. This data can be found here: https://www2.eecs.berkeley.edu/Research/Projects/CS/vision/bsds/.

## Author contributions

AC developed the methods, under the supervision of AP and BM. All authors contributed to the article and approved the submitted version.
